# A Novel Framework for Early Detection of Hypertension using Magnetic Resonance Angiography

**DOI:** 10.1038/s41598-019-47368-1

**Published:** 2019-07-31

**Authors:** Heba Kandil, Ahmed Soliman, Mohammed Ghazal, Ali Mahmoud, Ahmed Shalaby, Robert Keynton, Adel Elmaghraby, Guruprasad Giridharan, Ayman El-Baz

**Affiliations:** 1 Bioimaging Laboratory, Bioengineering Department, University of Louisville, Louisville, KY 40292 USA; 2 Computer Engineering and Computer Science Department, University of Louisville, Louisville, KY USA; 3 Faculty of Computer Science and Information, Information Technology Department, Mansoura University, Mansoura, 35516 Egypt; 4 Electrical and Computer Engineering Department, University of Abu Dhabi, Abu Dhabi, UAE

**Keywords:** Diagnosis, Translational research

## Abstract

Hypertension is a leading mortality cause of 410,000 patients in USA. Cerebrovascular structural changes that occur as a result of chronically elevated cerebral perfusion pressure are hypothesized to precede the onset of systemic hypertension. A novel framework is presented in this manuscript to detect and quantify cerebrovascular changes (i.e. blood vessel diameters and tortuosity changes) using magnetic resonance angiography (MRA) data. The proposed framework consists of: 1) A novel adaptive segmentation algorithm to delineate large as well as small blood vessels locally using 3-D spatial information and appearance features of the cerebrovascular system; 2) Estimating the cumulative distribution function (CDF) of the 3-D distance map of the cerebrovascular system to quantify alterations in cerebral blood vessels’ diameters; 3) Calculation of mean and Gaussian curvatures to quantify cerebrovascular tortuosity; and 4) Statistical and correlation analyses to identify the relationship between mean arterial pressure (MAP) and cerebral blood vessels’ diameters and tortuosity alterations. The proposed framework was validated using MAP and MRA data collected from 15 patients over a 700-days period. The novel adaptive segmentation algorithm recorded a 92.23% Dice similarity coefficient (DSC), a 94.82% sensitivity, a 99.00% specificity, and a 10.00% absolute vessels volume difference (AVVD) in delineating cerebral blood vessels from surrounding tissues compared to the ground truth. Experiments demonstrated that MAP is inversely related to cerebral blood vessel diameters (p-value < 0.05) globally (over the whole brain) and locally (at circle of Willis and below). A statistically significant direct correlation (p-value < 0.05) was found between MAP and tortuosity (medians of Gaussian and mean curvatures, and average of mean curvature) globally and locally (at circle of Willis and below). Quantification of the cerebrovascular diameter and tortuosity changes may enable clinicians to predict elevated blood pressure before its onset and optimize medical treatment plans of pre-hypertension and hypertension.

## Introduction

One in three adults in the US suffers from hypertension. Hypertension is a leading contributor of death in 410,000 patients in USA^1^. Many factors such as renal dysfunction, high sodium intake, and chronic stress contribute in the development of hypertension. The chronic elevation of cerebral perfusion pressure (CPP) changes the cerebrovasculature of the brain and disrupts its vasoregulation mechanisms. This cerebral vascular alteration has a severe effect on the human body organs^2^ and is a leading cause of cognitive impairment, strokes, dementia, ischemic cerebral injury, and brain lesions^3^. Specifically, recent studies hypothesized that changes in the cerebrovasculature and CPP precede the systemic elevation of blood pressure (BP)^4,5^.

Currently, sphygmomanometers are used to measure repeated brachial artery pressure to diagnose systemic hypertension after its onset. However, this method cannot detect cerebrovascular alterations that lead to adverse events which may occur prior to the onset of hypertension. Quantifying these cerebral vascular structural changes could help in predicting patients who are at a high risk of cerebral adverse events. This may enable early medical intervention prior to the onset of systemic hypertension, potentially mitigating vascular-initiated end-organ damage.

Previous studies have demonstrated vascular changes with hypertension. A direct relationship between cerebral microvascular changes and hypertension was demonstrated using ultra high-resolution magnetic resonance angiography (MRA) of the lenticulostriate arteries (LSAs)^6^. Chen *et al*. analyzed 3-D time-of-flight (TOF)-MRA and found that there is a significant decrease in the number of LSA stems in hypertension patients compared to normal people^7^. Cerebrovascular structural alterations such as changes in blood vessels’ diameters and tortuosity have been used in the diagnosis of many diseases. Cerebral blood vessels’ change in diameters has been reported as an early sign of cerebrovascular dysfunction from both *in-vivo* and clinical observations^8,9^. Vascular resistance in hypertension resulted from the reduction of lumen size of small size arteries and arterioles^10^. Carotid artery diameter change in rats has been correlated to the chronic elevation of BP^11^. Pulmonary hypertension in humans has been reported to correlate to changes of pulmonary arterial diameters^12,13^. Abnormal or excessive vascular tortuosity of blood vessels has also been clinically observed to precede the onset of multiple vascular and non-vascular diseases^14,15^. Vascular tortousity has been linked to hypertension, genetic defects, aging, atherosclerosis, and diabetes mellitus^14^. Tortuosity has been used to measure how sharply a vessel is twisting or bending. Tortuosity of retinal blood vessels has been used and assessed by ophthalmologists as a diagnostic parameter^16–18^. Increase of tortuosity of coronary vessel was linked to patients with hypertension^19^. Hemispheric white matter tortuosity has been correlated to the severity of systemic hypertension and elevated cerebral perfusion pressure^20^. Thus, detection and quantification of cerebral vascular changes in diameters and tortuosity at an early stage would help clinicians in early diagnosis and identification of patients at a risk of hypertension, and initiating a treatment before the onset of the disease.

However the widespread usage of imaging technologies such as magnetic resonance angiography, alterations or remodeling of cerebral blood vessels’ diameters and tortuosity have not been correlated to elevated arterial pressure due to limitations associated with current segmentation algorithms which cannot delineate small blood vessels efficiently. Manual segmentation of blood vessels is time consuming and intensive, error-prone, and is subject to inter-observer variability. In addition, semi-automatic blood vessel segmentation algorithms may need further investigation, revisions and/or evaluations by clinicians. In this manuscript, a novel framework is presented to automatically segment and accurately measure and quantify cerebrovascular changes using MRA data, and correlate these changes to mean arterial pressure (MAP). The framework includes a proposed novel automatic local adaptive segmentation algorithm which was capable of delineating both large as well as small blood vessels from MRA data. To the best of our knowledge, this study is the first to investigate the cerebrovasculature changes that *precede* hypertension from MRA.

## Methods

**Figure 1:**
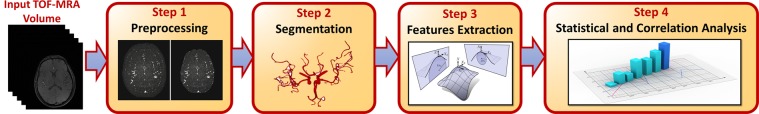
A framework for detection and quantification of cerebral vascular changes.

The proposed framework (Fig. 1) includes 3 basic modules: 1) a novel, 3-D fully-automated local adaptive segmentation algorithm that extracts large as well as small cerebral blood vessels accurately, 2) a feature extraction module where imaging markers are quantified to predict the potential of elevated blood pressure, and 3) a statistical and correlation analysis module to correlate MAP to cerebrovascular change. These three modules are explained in more details in the following subsections.

### A 3-D Local adaptive segmentation algorithm

Segmentation is an essential step in most of the medical imaging analysis systems. Segmentation accuracy is affected by many factors such as scanning parameters, application domain, and imaging modality. Particularly, segmentation of cerebral blood vessels from MRA data has several challenges including the complex nature of the vasculature, diameter and density of small vessels, dynamic range of intensities, acquisition errors, noise, and high inter- person variability of the vascular tree which hinders the creation of common atlas to be used for segmentation, as done for other human organs. Therefore, there is a limitation of current segmentation algorithms to delineate cerebral blood vessels efficiently, specifically smaller ones.

#### Skull stripping

**Figure 2:**
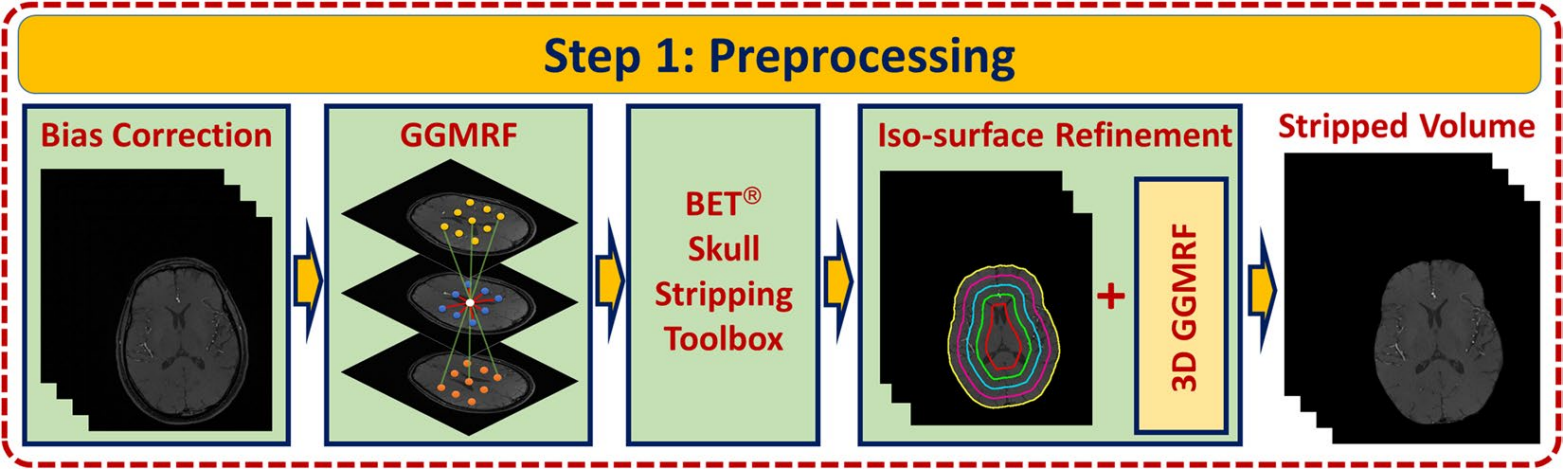
Steps of Preprocessing Stage.

A preprocessing step (Fig. 2) precedes the segmentation step to account for any biasing or inhomogeneity of the MRA data. A nonparametric bias correction algorithm^21^ was used to reduce any effects of noise and remove data inconsistencies. Then, a homogeneity enhancement algorithm employing a 3-D generalized Gauss-Markov random field (GGMRF) model^22^ was used. It makes use of the 3-D spatially homogeneous pairwise interactions of the 26-neighborhood system and minimizes differences between a centered voxel and its 26 neighbors using the following energy function:

1 $$\widehat{{q}_{s}}={\rm{\arg }}\,\mathop{{\rm{\min }}}\limits_{{\tilde{q}}_{s}}[|{q}_{s}-{\tilde{q}}_{s}|+{\rho }^{\alpha }\,{\lambda }^{\beta }\,\sum _{r\varepsilon {v}_{s}}\,{\eta }_{s,r}|{\tilde{q}}_{s}-{q}_{r}{|}^{\beta }]$$

such that *q*_*s*_ and ${\tilde{q}}_{s}$ represent original and new estimated gray-levels; *v*_*s*_ represents the 26-neighborhood system; ${\eta }_{s,r}$ represents the GGMRF potential; *λ* and $\rho \,$ represent the scaling factors; $\alpha \,\varepsilon \,\{1,2\}$ was used to define the prior distribution of the estimator ($\alpha =1$ for Laplace or $\alpha =2$ for Gaussian); and *β* was used in controlling the smoothing level such that $\beta \,\varepsilon \,[1.01,2.0]$ ($\beta =2$ for smoothing and $\beta =1.01$ for relatively abrupt edges). A skull stripping procedure was utilized and applied on the preprocessed data to remove human brain’s fat tissues that have a similar visual appearance to that’s of blood vessels and retain brain tissues only. It combines Markov-Gibbs random field (MGRF) model of data with a geometric deformable model (brain isosurface) which preserves the cerebral topology during the process of extraction. Algorithm 1 presents the details of skull stripping algorithm.

#### A Linear Combination of Discrete Gaussians (LCDG)-based segmentation

**Figure 3:**
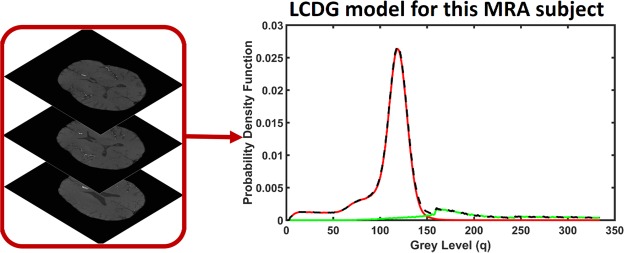
Empirical density normalization using the LCDG model.

A Bayesian framework was used to extract an initial vasculature where a linear combination of discrete Gaussians (LCDG)^23^ was used for estimating marginal probability density of MRA voxel values for cerebral vessels and other cerebral tissues (Fig. 3). The LCDG model used has *C*_*p*_ positive and *C*_*n*_ negative components for cerebral vessels and other tissues and is given by the following equation:

2 $${p}_{w,{\rm{\Theta }}}(q)=\sum _{r=1}^{{C}_{p}}\,{w}_{p,r}\psi (q|{\theta }_{p,r})-\sum _{l=1}^{{C}_{n}}\,{w}_{n,l}\psi (q|{\theta }_{n,l})$$

where $${{\rm{\Phi }}}_{\theta }(q)$$ is the cumulative Gaussian function with $$\theta =(\mu ,{\sigma }^{2})$$ for the mean, *μ*, and variance, *σ*^2^ such that $$\psi (q|\theta )={{\rm{\Phi }}}_{\theta }(q+0.5)-{{\rm{\Phi }}}_{\theta }(q-0.5))$$ for $$q=1,\ldots ,Q-2,\psi (0|\theta )={{\rm{\Phi }}}_{\theta }(0.5)$$ and $$\psi (Q-1|\theta )=1-$$$${{\rm{\Phi }}}_{\theta }(Q-1.5)$$. Total summation of the non-negative weights of the LCDG models is equal to one. For detailed description of the LCDG model, see^23,24^. Parameters of the LCDG model (prior probability, number of Gaussian components, mean, and variance) were estimated using the modified Expectation Maximization (EM) algorithm^23^. Finally, the extraction of the blood vessels was performed based on the following Bayesian rule: $$P(v)p(q|v)\ge P(O)p(q|O)$$. Where *P*(*v*) is the prior probability of the cerebral blood vessels, and *P*(*O*) is the prior probability of the other cerebral tissues.

#### Blood vessels’ segmentation refinement

**Figure 4:**
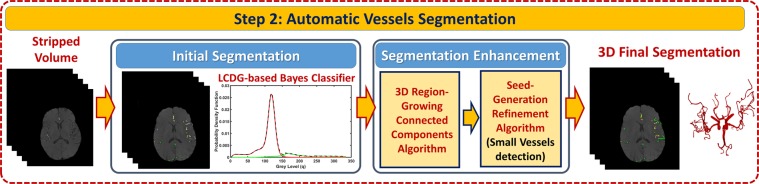
Steps of Segmentation Stage.

The initial segmentation of the vasculature may miss some of the small blood vessels. To handle this challenge, a novel 3-D local adaptive segmentation algorithm has been developed (Fig. 4). The algorithm processes the initially segmented vasculature to find more blood vessels, specifically the smaller ones. In this algorithm, each slice was divided into a set of connected components. A search window of adaptive size was then centered around every component in the set where a new separation threshold was estimated as $T=\frac{{\mu }_{b}+{\mu }_{o}}{2}$, where *μ*_*b*_ represents average intensity of blood vessels and *μ*_*o*_ represents average intensity of other cerebral tissues. A novel seed-generation refinement algorithm was developed and utilized to detect seeds within regions that have a high potential to contain smaller vessels missed in the initial segmentation. A 3-D region growing connected components algorithm was subsequently used to delineate the final, connected vasculature. Steps of the segmentation algorithm are presented in Algorithm 2.

### Cerebral vascular system feature extraction

#### Median vascular radius

**Figure 5:**
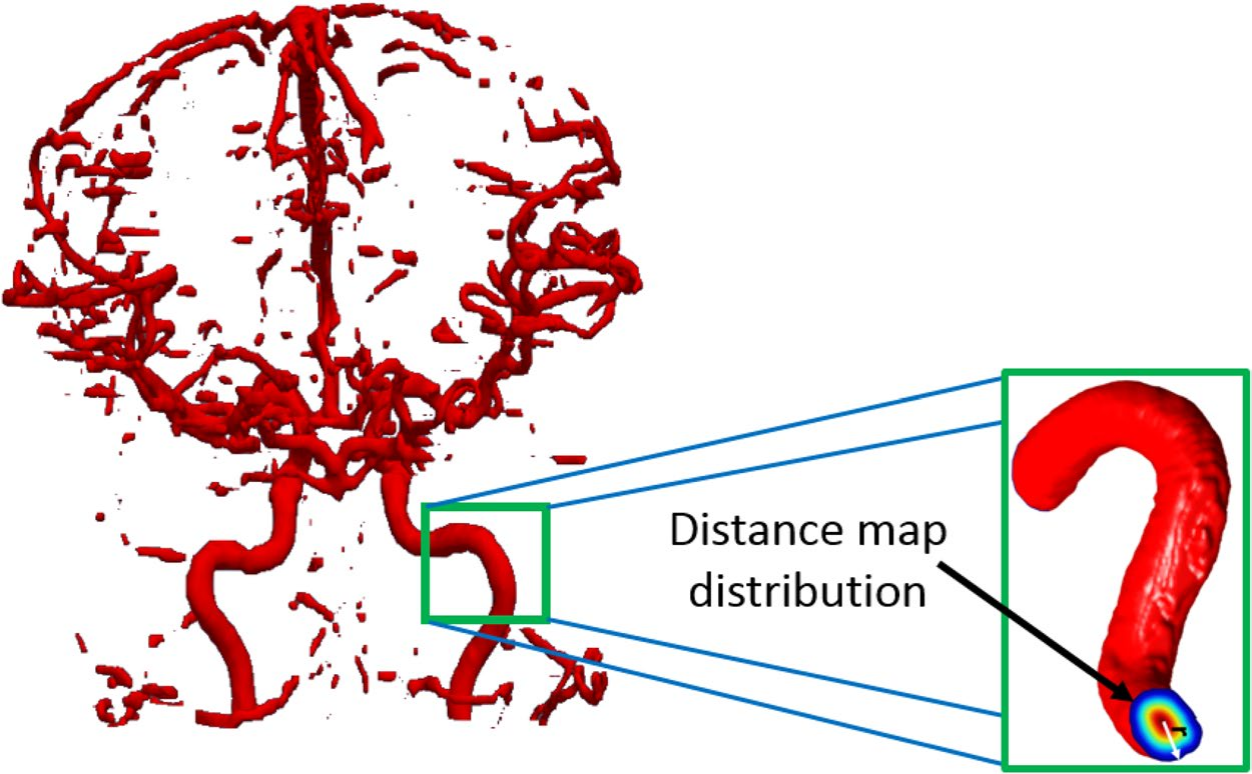
Visualization of the distance map calculated for each blood vessel.

For each subject, medians of vascular radii were obtained by estimating distance map for the delineated vascular tree. Making use of these measurements, the cumulative distribution function (CDF) of the vascular radii was estimated as the cumulative distribution of the PDF. The CDF *F*_*X*_ of a discrete random variable *X* is obtained as $F(x)=P(X\le x)={\sum }_{t\le x}\,f(t)$. The CDF provides a probability estimate for the blood vessels the exists at or below a specific vascular diameter point. Each CDF value represents the average of the vascular diameters in the MRA volume (Fig. 5).

**Algorithm 1:**
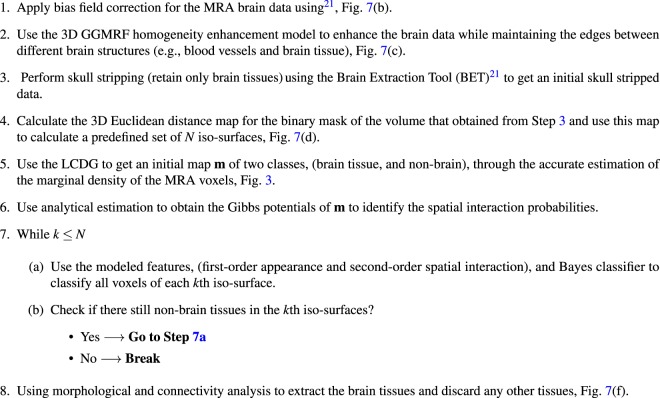
Brain tissue extraction from MRA scans.

**Algorithm 2:**
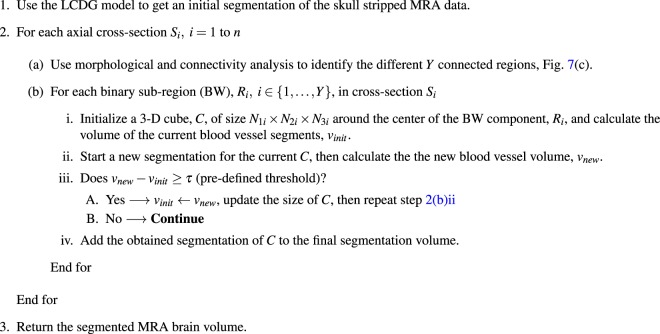
Local Adaptive Segmentation.

#### Tortuosity of cerebral blood vessels

Tortuosity from imaging modalities can be measured either as the ratio of a vessel curve length over the line distance between the two ends^19,25,26^, or as the cumulative sum of angles between segment vectors normalized by vessel length (total curvature or mean curvature)^26–28^. Gaussian and mean curvatures are considered to be the most significant types of curvatures in surface theory^29^. Thus, in the proposed framework, tortuosity was measured from MRA data by calculating mean and Gaussian curvatures across the whole vasculature of each patient. Mean curvature equals $({k}_{1}+{k}_{2})$/2, where *k*_1_, *k*_2_ are the principal curvatures, and is an extrinsic measure of curvature that depends on the embedding. Gaussian curvature equals ${k}_{1}\times {k}_{2}$ and is an intrinsic property of the surface and does not depend on the embedding of the surface.

**Figure 6:**
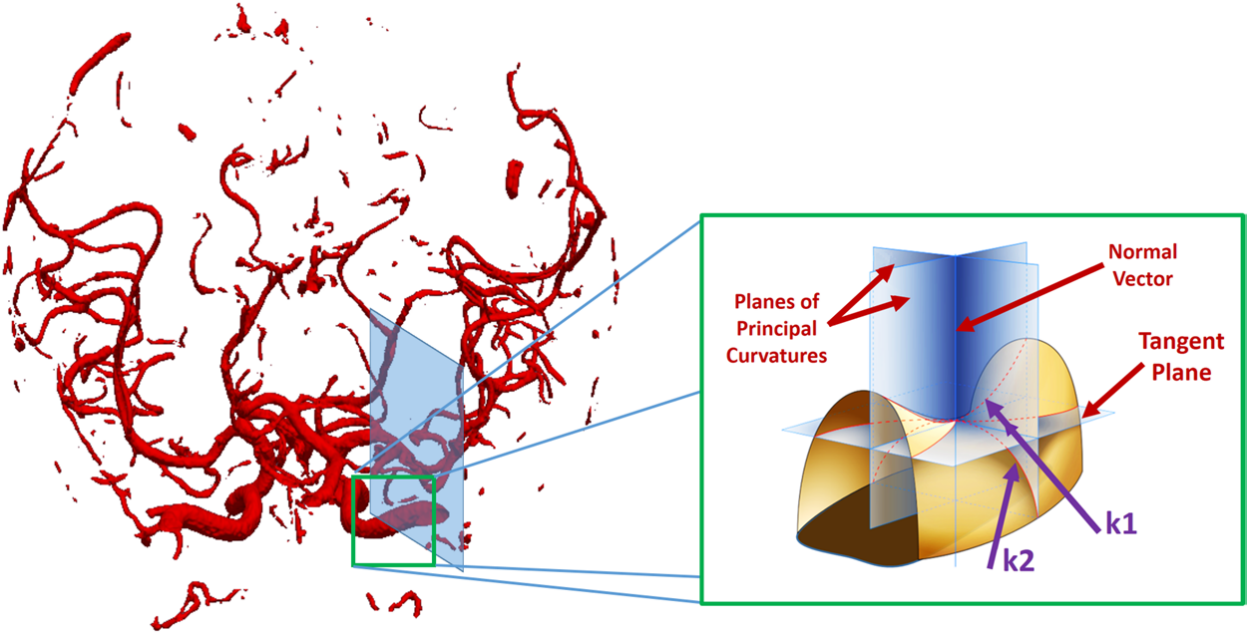
Visualization of the calculation of curvatures. N is the normal vector, and k1, k2 are the principal curvatures (maximum and minimum normal curvatures).

The surface of the segmented vascular tree was modeled as a triangulated mesh. Following the methodology proposed by Smedby *et al*.^30^, and generalizing to higher dimensions, we defined tortuosity as the integral of absolute curvature |*K*|. Total (or Gaussian) curvature K was estimated in the neighborhood of each mesh vertex using the algorithm of Chen and Schmitt^31^. Briefly, let *v*_*i*_ be the coordinates of vertex i, *n*_*i*_ is the surface normal (a weighted average of normals to the triangles incident on i), and *N*_*i*_ is a vertex neighborhood of i, specifically the one-ring neighborhood if it contains at least four neighbors, or two-ring neighborhood in other cases. Then for each vertex j in *N*_*i*_, let ${e}_{ij}={v}_{j}-{v}_{i}$, and ${t}_{ij}=\frac{{e}_{ij}-({n}_{i}.{e}_{ij}){n}_{i}}{\parallel {e}_{ij}-({n}_{i}.{e}_{ij}){n}_{i}\parallel }$; the finite-difference approximation of normal curvature at *v*_*i*_ in the direction *t*_*ij*_ is ${k}_{ij}=-\,\frac{{e}_{ij}({n}_{j}-{n}_{i})}{\parallel {e}_{ij}{\parallel }^{2}}$. Euler’s theorem states that the normal curvature along tangent direction t is $k(\theta )={k}_{1}{co}{{s}}^{2}\theta +{k}_{2}{si}{{n}}^{2}\theta$, where *θ* is the angle t makes with the first principal direction *E*_1_, and *k*_1_ and *k*_2_ (principal curvatures), with ${k}_{1}\ge {k}_{2}$. Given a sample of tangent vectors and corresponding normal curvatures, the Chen-Schmitt algorithm estimates principal directions and curvatures. The total curvature $K={k}_{1}\times {k}_{2}$ can then be computed (Fig. 6).

### Materials and procedure

Magnetic resonance angiography scans and blood pressure measurements used in this study were collected from 15 participants (Female = 7, Male = 8, Age = 49.2 ± 7.3 years) during a study period that extended for 700-days (day 0 (*t*_0_) and on day 700 (*t*_1_)). The total number of data sets processed was 30. Data were collected and approved by the Institutional Review Board at the University of Pittsburgh and the research was performed in accordance to the relevant guidelines and regulations. Participants of ages 35–60 years were enrolled in the study with the following exclusion criteria: 1) general medical conditions: ischemic coronary artery disease, pregnancy, chronic liver disease, cancer (treatment < 12 months), diabetes mellitus (fasting blood glucose > 125 mg/dL), or chronic kidney disease (creatinine > 1.2 mg/dL); 2) neuropsychiatric conditions: multiple sclerosis, stroke, epilepsy, serious head injury, brain tumor, and major mental illness; 3) using prescription medications for hypertension and psychotropic drugs. Participant were selected to be pre-hypertensive with a systolic BP > 120 and <140 mmHg or a diastolic BP > 80 and <90 mmHg.

Initial screening via phone calls was made with each participant to ensure eligibility. All participants provided informed consent before any study procedures. Participants had to attend to the lab three times within two weeks. During the first visit, participants provided their medical history, blood pressure readings, some physical traits such as weight, height, etc. (Table 1). In the second visit, blood pressure readings were taken from the participants, followed by a 2-hour neuropsychological battery of tests. The third visit included a 1-hour MRI screening.

**Table 1:** Participant Demographics and Characteristics.

Patient	Age	Gender	Race	BMI	SBP1	DBP1	SBP2	DBP2	MAP(pre)	MAP(post)
1	50	1	1	32.97	121.5	85.5	118.5	69	97.5	85.5
2	41	1	1	27.59	125.5	88.5	129.5	93	100.8	105.2
3	59	1	2	23.32	130.5	82.5	120.5	78	98.5	92.2
4	35	2	1	42.77	118	80.5	105.5	69	93	81.2
5	48	1	1	30.64	130.5	83	143.5.5	94	98.8	110.5
6	53	2	2	22.77	121	88.5	130.5	100.5	99.3	110.5
7	59	2	1	25.70	120	80.5	103.5	66.5	93.7	78.8
8	58	1	1	28.43	129	78.5	134.5	86	95.3	102.2
9	50	1	2	25.53	114	84.5	120	88	94.3	98.7
10	54	2	2	28.31	119	84.5	115.5	76	96	89.2
11	53	2	2	28.40	124.5	73	101	69	90.2	79.7
12	55	1	2	33.83	133.5	85	123.5	79.5	101.2	94.2
13	50	2	1	31.79	124	81.5	106.5	70.5	95.7	82.5
14	36	1	2	39.33	105.5	85	132.5	95.5	91.8	107.8
15	46	2	2	28.55	124	81.5	108.5	74.5	95.7	85.8

Gender (1 = male, 2 = female), Race (1 = white, 2 = black), BMI: body mass index, SBP1 and SBP2: systolic blood pressure at *t*_0_ and *t*_1_ respectively, DBP1 and DBP2: diastolic blood pressure at *t*_0_ and *t*_1_ respectively, MAP(pre) and MAP(post): MAP at *t*_0_ and *t*_1_ respectively.

Blood pressure measurements were obtained using the auscultatory technique with cuff size appropriate to patient arm after giving him/her a seated rest of 5-minutes. Two measurements were taken with at least 1-minute separation time. This procedure was repeated on a second day and the average of the four readings taken during both visits was calculated and used to calculate MAP value. Participants were invited to the follow-up assessment after approximately 2 years where the blood pressure measurements and the MRA scans were obtained again. Average of blood pressure readings on *t*_0_ was 122 ± 6.9 mmHg systolic and 82 ± 3.8 mmHg diastolic, while on *t*_1_, average was 118.9 ± 12.4 mmHg systolic and 79.9 ± 11.0 mmHg diastolic. Blood pressure measurements of patients were comparable over time, but measurements for each patient changed or stayed the same temporally.

MRA scans were collected by a 3 T Trio TIM scanner with a 12-channel phased-array head coil. Each scan was composed of 3-D multi-slab high-resolution images with about 160 slices, a thickness of 0.5 mm, a resolution of 384 × 448, a flip angle of 15 degrees, a repetition time of 21 ms, and an echo time of 3.8 ms. MRA data were analyzed blinded to patients’ blood pressure.

## Results

### Segmentation and statistical analysis

The proposed automatic novel adaptive segmentation algorithm was evaluated using commonly used segmentation evaluation metrics; Dice similarity coefficient (DSC), the absolute vessels volume difference (AVVD), sensitivity, and specificity. The first metric is the DSC, one of the most commonly used similarity metrics, that characterizes the agreement between the segmented (**S**) and the gold standard (**G**) regions based on the determination of true positive (*TP*) value, true negative (*TN*) value, false negative (*FN*) value, and false positive (*FP*) value. The *TP* is defined as the number of positively labeled voxels that are correct; the *FP* is the number of positively labeled voxels that are incorrect; the *TN* is the number of negatively labeled voxels that are correct; and the *FN* is the number of negatively labeled voxels that are incorrect. These values are used to calculate the DSC as described in details in^32^: The calculated value of the DSC can have a percentage value in the range 0% to 100%, where 0% means strong dissimilarity and 100% means that there is a perfect similarity. To obtain the gold standard that was used in the segmentation evaluation process, an MRA expert delineated the brain vessels. The second used evaluation metric is AVVD, an area-based metric, which measures the volume difference (percentage) between the output of the segmentation framework, **S**, and the gold standard, **G**, as follows:

3 $${\rm{AVVD}}({\bf{G}},{\bf{S}})=\frac{|{\bf{G}}-{\bf{S}}|}{|{\bf{G}}|}$$

where |**G** − **S**| is the absolute difference between the number of voxels in **G** and **S**, |**G**| is the number of voxels in **G**. Moreover, both the sensitivity, $(Sens=\frac{TP}{TP+FN})$, and specificity, $(Spec=\frac{TN}{TN+FP})$ of the segmentation have been evaluated to measure both the true positive and true negative detection accuracy.

**Figure 7:**
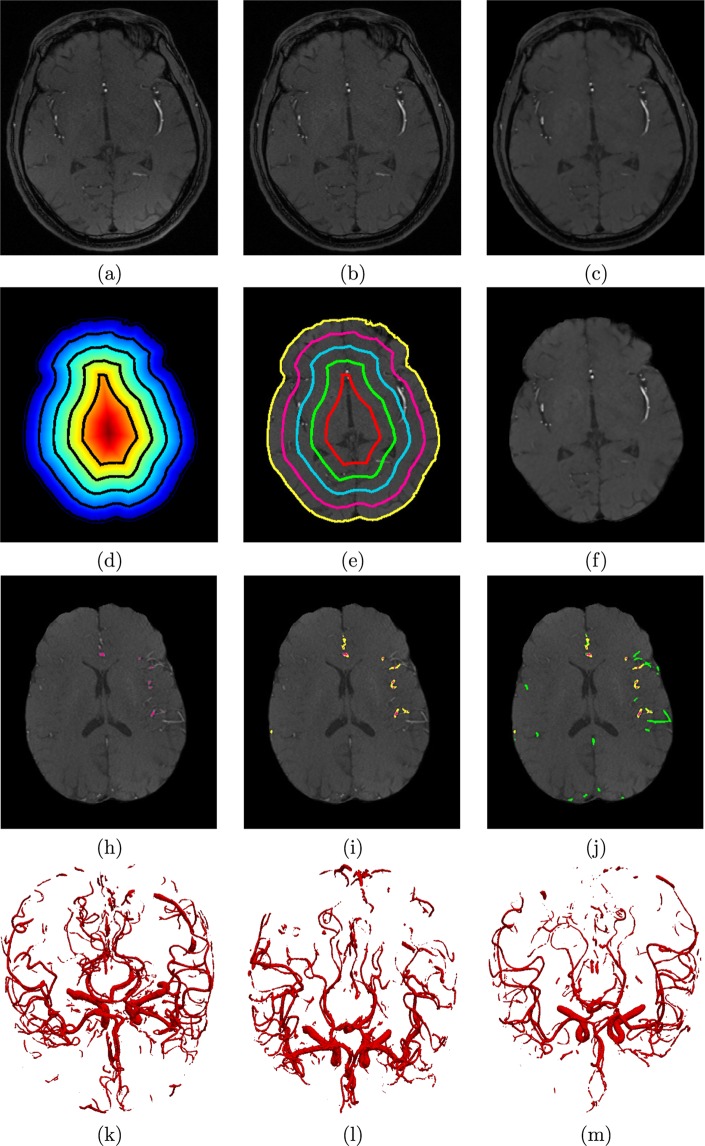
Sample output of the local adaptive segmentation algorithm: (**a**) Original (raw) image, (**b**) After bias-correction, (**c**) After GGMRF-enhancement, (**d**) Distance map and iso-surfaces generated, (**e**) Subsurfaces-based extraction of brain tissues, (**f**) Final delineated cerebrovasculature (**h**) Initial LCDG global segmentation, (**i**) Results after applying the proposed local adaptive segmentation from the same plane, (**j**) and preceding and succeeding planes, and 3-D visualization of results using a growing tree model (**k**–**m**).

**Figure 8:**
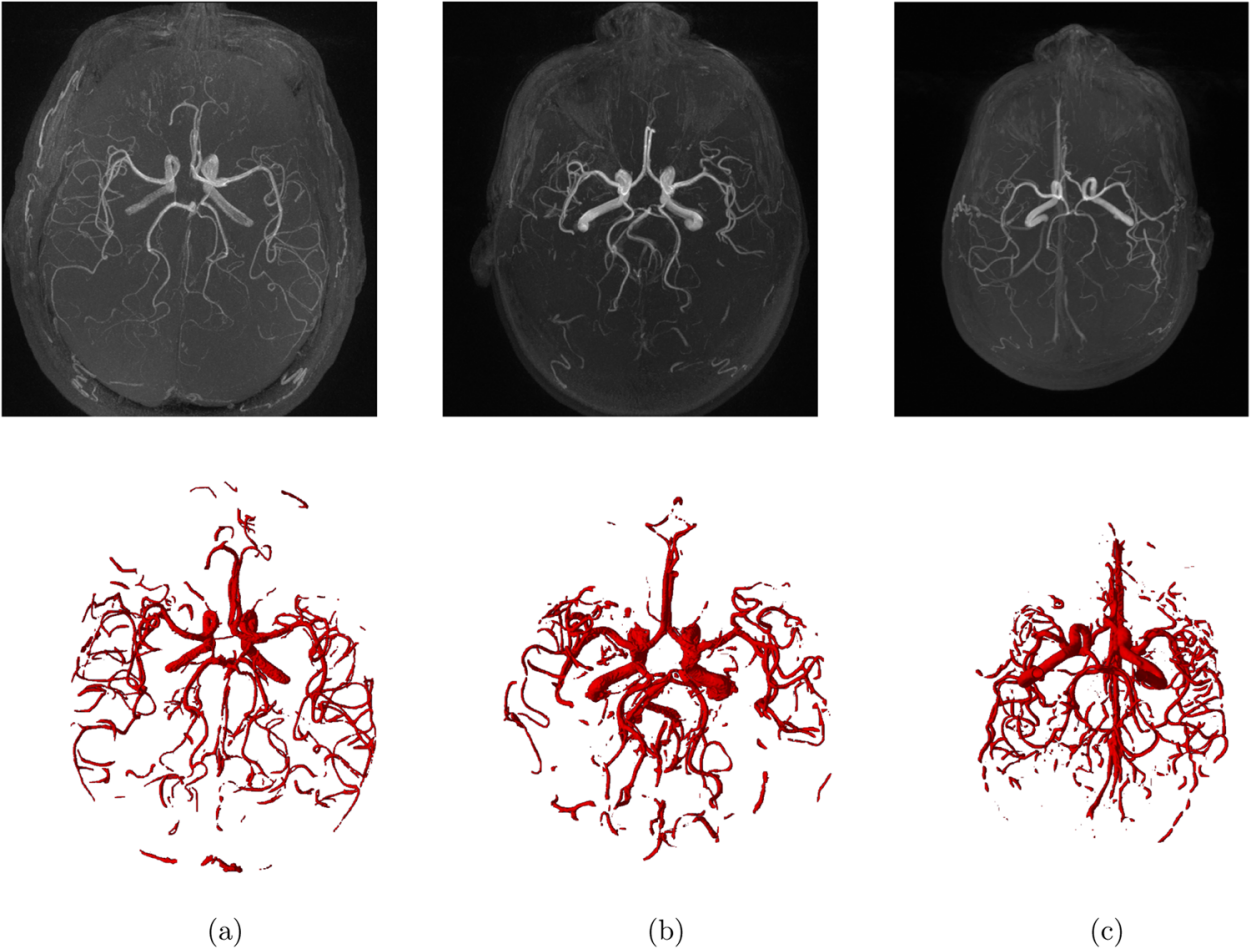
Segmentation results for three MRA subjects (**a**–**c**). The maximum intensity projection (MIP) (first-row); 3D segmentation obtained by the proposed segmentation approach (second-row).

The proposed automatic novel adaptive segmentation algorithm obtained a DSC of ~92.23%, a sensitivity of ~94.82%, a specificity of ~99.00%, and an AVVD of ~10.00% in delineating cerebral blood vessels compared to the manually segmented ground truth. These results demonstrate the high accuracy and efficacy of this algorithm. Figure 7 shows an output instance of the segmentation algorithm. To highlight the accuracy enhancement of the proposed approach over existing methods, a comparison to the global statistical approach^24^ was conducted and the results are shown in Table 2. Figure 8 shows sample 3-D segmentation results for three MRA subjects along their maximum intensity projection (MIP). These qualitative results demonstrate how the proposed segmentation approach is capable of obtaining fine details of the brain vasculature.

**Table 2:** Result of comparing the proposed segmentation algorithm and the Global Statistical Based approach (GSB)^24^ in terms of the Sensitivity, Specificity, DSC and AVVD.

Method	Sensitivity	Specificity	DSC, %	AVVD, %
Proposed	94.82%	99.00%	92.2311	10.03
GSB	85.28%	97.50%	80.12	23.62

R-software version 3.2 was utilized to perform statistical analysis. The add-on package lme4^33^ was used to study the potential correlation between blood pressure measurements and MRA data. MAP dependence upon features of the cerebral vasculature was tested using a mixed-effects linear model of fixed effects of median of vascular radius (represents blood vessels diameters) and averages and medians of both Gaussian curvature and mean curvature of cerebral blood vessels (represent tortuosity of blood vessels). A random intercept per patient was also included in the test model. This analysis defined MAP using the equation that follows, ${\rm{MAP}}=(2\,\ast \,{\rm{Diastolic}}\,{\rm{BP}}+{\rm{Systolic}}\,{\rm{BP}})/3$. For each feature quantified, we tested our framework both globally (over the whole brain) and locally (brain was segmented into upper section (above circle of Willis), and lower section (circle of Willis and below)). Correlation analysis was performed using MATLAB R2017a software.

Results of statistical experiments demonstrated a statistically significant (p-value < 0.05) inverse correlation between MAP and alterations in diameters of blood vessels (represented by median of vascular radius) globally over the whole brain (Table 3) and locally at the lower section of the brain (Table 4). Additionally, the statistical analysis demonstrated that MAP was significantly correlated with median of mean curvature, median of Gaussian curvature, and average of mean curvature, (p-value < 0.05) globally (Table 3) and locally at the lower section of the brain (Table 4). In the upper section of the brain (above the circle of Willis), the p-values for median of mean curvature, median of Gaussian curvature, average of mean curvature and average of Gaussian curvature were 0.068, 0.063, 0.060, 0.027 respectively.

**Table 3:** MAP Response on Change of Tortuosity and Diameter of Blood Vessels Globally.

Variable (feature)	Chi-Squared (*χ*^2^) (Df = 1)	p-value
*Median of Mean curvature*	4.0297	0.04
*Median of Gaussian curvature*	4.1731	0.04
*Average of Mean curvature*	4.3503	0.037
*Median of vascular radius*	4.1022	0.04

**Table 4:** MAP Response on Change of Tortuosity and Diameter of Blood Vessels Locally.

Variable (feature)	Chi-Squared (*χ*^2^) (Df = 1)	p-value
*Median of Mean curvature* (*lower*)	4.072	0.04
*Median of Gaussian curvature* (*lower*)	3.9889	0.04
*Average of Mean curvature* (*lower*)	4.5309	0.03
*Median of vascular radius* (*lower*)	4.6443	0.03

## Discussion and Limitations

It is common that patients with hypertension are asymptomatic, sometimes even in advanced stages. Even a measurement of high blood pressure is often neglected as a temporary result due to stress or other factors rather than chronic hypertension. Predicting the potential of developing a hypertension in early stages may help in slowing the progression of the disease by following proactive and preventive lifestyles recommended by clinicians. Timely information regarding vascular health would potentially enhance the quality of life for patients and their families and reduce the health care costs. In this study we presented a framework that would help clinicians in predicting elevated blood pressure before its onset. The proposed automatic local adaptive segmentation algorithm was able to rapidly delineate large and small cerebral blood vessels with high degrees of specificity and sensitivity. Importantly, the 3-D segmentation algorithm is fully automatic and is applicable to both healthy, and non-healthy vessels. Previous methodologies published in literature are suitable only for healthy vessels due to inherent assumptions that do not fit with pathological vessels such as linearity and circular cross-section^34^. Studies that developed automatic cerebrovasculature segmentation algorithms have been reported^35–38^. For example, one study proposed an architecture based on using a deep convolutional neural network (CNN) to automatically segment cerebral blood vessels from TOF-MRA datasets of healthy subjects by training the delineated manually annotated data. Their proposed framework was able to delineate cerebral blood vessels with a DSC ranging from 0.764 to 0.786^35^. A deep learning approach called DeepVesselNet was proposed in^36^ where a 3-D CNN architecture was employed to segment blood vessels along with other tasks such as vessels center-lines prediction and bifurcations detection. In their methodology, they used cross-hair filters (one of the components of DeepVesselNet) from three intersecting 2-D filters to help in avoiding memory and speed problems of traditional 3-D networks while at the same time taking advantages of the 3-D information in volumetric data. Their experiments showed that their method performed well compared to 3-D filters and at the same time improved speed and memory consumption significantly. Another MRA- based vasculature segmentation method was proposed in^37^ where they first performed background subtraction and vessel reservation by applying volume projection, 2-D segmentation, and back-projection operations. Then, they utilized a stochastic expectation maximization algorithm to estimate the PDF of remaining vessels’ voxels, which were assumed to be mixture of one Rayleigh and two Gaussian distributions. Their method classified image voxels into three classes; background, middle region, and vascular structure. Subsequently, the K-means method, which is based on the gradient of remaining vessels was utilized to detect true positives around boundaries of vessels effectively. The methodology could achieve accurate segmentation in regions of low contrast. However, one disadvantage of their method was the computing time consumed by the K-means method to determine the appropriate gradient value. In contrast, the proposed segmentation algorithm utilizes an adaptive thresholding methodology and overcomes the limitations associated with MRA images such as biasing, noise, or resolution problems, which enables efficient extraction of small and large cerebral blood vessels. Importantly, the automatic nature of this framework ensures that there will be no intra- or inter-observer variability because there are no human interactions.

**Figure 9:**
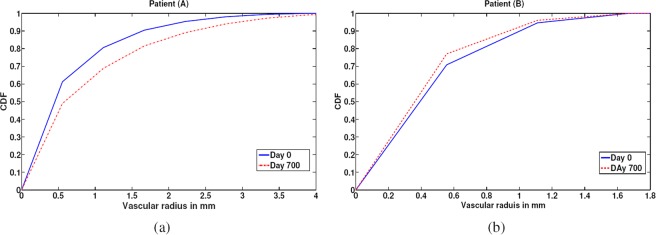
CDF estimations and the corresponding medians of vascular radii at *t*_0_ and *t*_1_ for subjects A, B. (**a**) CDF saturated at a vascular radius of smaller value on *t*_0_ compared to *t*_1_ for Subject A. (**b**) CDF saturated at a vascular radius of smaller value for Subject B on *t*_1_ compared to *t*_0_.

**Figure 10:**
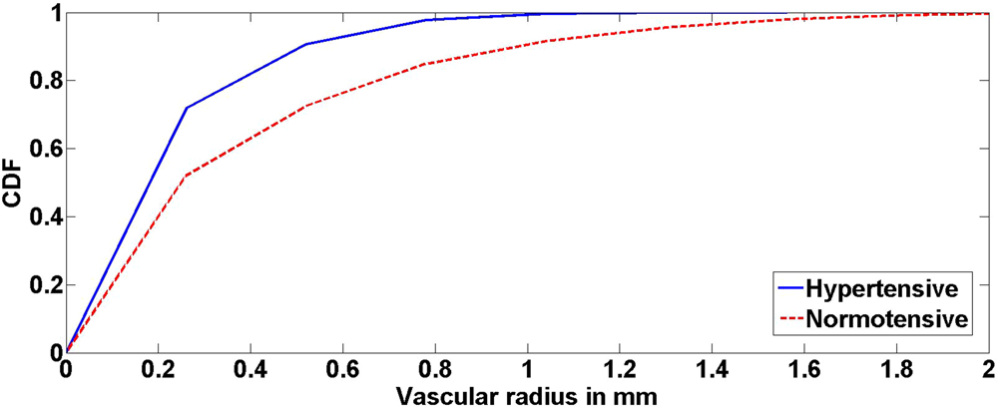
CDF estimations and the corresponding median vascular radii for a hypertensive subject and a normotensive subject. Elevated blood pressure corresponded to CDF saturation at a smaller vascular radius value. These results support the efficacy of the presented framework.

This study demonstrated that changes of cerebral blood vessel diameters were inversely correlated to MAP globally (over the entire brain) as well as at and below the circle of Willis (lower section of the brain). Cerebral blood vessels below the circle of Willis are typically larger compared to blood vessels in the upper compartment of the brain. While the segmentation algorithm can delineate these smaller blood vessels, changes in the small blood vessel diameters were not statistically significant. This is potentially due to the smaller variations in diameters of small blood vessels that is limited by the resolution of the MRA images. Our proposed approach used CDF, which is a measure of diameter over the entire volume of the brain rather than the diameter of a vessel at a specific point. Thus, CDF was used to represent the median radius of the vessels globally, simplifying temporal comparisons and facilitating analysis. As an example, Fig. 9 presents the temporal alterations in radii of blood vessels and CDFs for two different subjects A and B. MAP values for subject A were decreasing from *t*_0_ to *t*_1_, while MAP values for subject B were increasing from *t*_0_ to *t*_1_. As shown in Fig. 9, the CDF for subject A saturated (CDF reaches 1) at a vascular radius of smaller value on *t*_0_ (higher MAP) compared to *t*_1_. On the contrary, the CDF for subject B saturated at a vascular radius of smaller value on *t*_1_ (higher MAP) compared to *t*_0_ (Table 5). The given results supported the efficacy of the presented framework to distinguish intra-patient temporal alterations in cerebrovasculature and demonstrated an inverse relationship between MAP and vascular diameters. Importantly, the framework recognized temporal alterations in cerebral vascular system in response to MAP in non-hypertensive patients (MAP < 120 mmHg). Thus, the presented framework would help ascertain risk of cerebral vascular events or systemic hypertension before its onset. Figure 10 illustrates median vascular radii of a hypertensive and normotensive subjects and their corresponding CDFs. The CDF of the hypertensive subject saturated at a vascular radius of smaller value (solid line), compared to the normotensive subject (dotted line). Additionally, the median vascular radius of the hypertensive subject was 0.46 mm smaller compared to the normotensive subject demonstrating the capability of the framework to discern inter-patient variabilities.

**Table 5:** Measurements of Blood Pressure of Subjects A, B.

Subject	Day 0 (*t*_0_)			Day 700 (*t*_1_)		
	Systolic BP	Diastolic BP	MAP	Systolic BP	Diastolic BP	MAP
**A**	124.5 mmHg	73 mmHg	90.17 mmHg	101 mmHg	69 mmHg	79.67
**B**	125.5 mmHg	88.5 mmHg	100.83 mmHg	129.5 mmHg	93 mmHg	105.17

**Figure 11:**
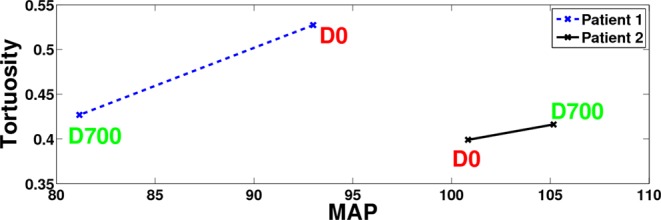
A sample of two patients to explain the correlation between Tortuosity and Mean Arterial Pressure (MAP). Patient 1 shows a decrease in tortuosity index from *t*_0_ to *t*_1_ corresponding to a decrease in MAP value for this patient. Patient 2 shows a slight increase from *t*_0_ to *t*_1_ corresponding to an increase in MAP value for this patient. Hence, these results show that MAP is directly correlated to tortuosity of cerebral blood vessels.

In the proposed framework, blood vessels’ tortuosity was measured by the calculation of mean and Gaussian curvatures. Algorithms proposed in literature have typically used a 2-D methodology for estimating curvatures. In contrast, our framework presents a novel 3-D methodology to estimate curvatures by using a 3-D mesh to model the vasculature obtained from segmentation. This three dimensional approach results in a more accurate representation and calculation of tortuosity in the cerebral volume. Our study demonstrated that change in cerebrovascular tortuosity was strongly correlated to MAP (Fig. 11) globally and in the lower section of the brain. In the upper section of the brain, the correlation values were not statistically significant due to the limited clinical sample size and smaller tortuosity changes observed in small diameter blood vessels. However, the correlation was trending towards statistical significance and additional data collection is currently underway. In summary, the results of this study is supportive of previously published hypothesis that cerebrovascular tortuosity changes may precede hypertension^6,7,14,15,19,20^.

A limitation of our study is that we had temporal MRA data from a small sample size of 15 patients. To the best of our knowledge, there are no free standard databases available in this field. Despite the small sample size, the cerebrovascular changes were adequate to enable statistical significance. The efficacy of the proposed framework to detect and quantify cerebrovascular structural changes could potentially enable clinicians in formulating appropriate medical treatment plans to mitigate risks of adverse events. Additionally, the framework may enable better follow-up of patients and to test the effectiveness of the treatment regimen in slowing down the progression of cerebrovascular changes. However, despite the accuracy of the framework in quantifying cerebrovascular changes from MRA, our approach may be limited to patients with high risk of hypertension or adverse events due to cost considerations. While MRA is expensive, the cost hypertension medication alone is $ 2000/year per patient and the hospitalization cost for a hemorrhagic stroke is over $ 32,000^39,40^. Thus, MRA screening may be cost effective long-term especially in patients with high risk of developing hypertension. Access to MRA, while limited in remote rural areas, is readily available in major care centers and hospitals that serve rural areas in the US.

## Data Availability

Materials, data, and associated protocols will be available to readers after the manuscript is being accepted.
